# Antibiotic-Antiapoptotic Dual Function of *Clinacanthus nutans* (Burm. f.) Lindau Leaf Extracts against Bovine Mastitis

**DOI:** 10.3390/antibiotics9070429

**Published:** 2020-07-21

**Authors:** Aussara Panya, Hataichanok Pundith, Supawadee Thongyim, Thida Kaewkod, Thararat Chitov, Sakunnee Bovonsombut, Yingmanee Tragoolpua

**Affiliations:** 1 Department of Biology, Faculty of Science, Chiang Mai University, Chiang Mai 50200, Thailand; hataichanok064@gmail.com (H.P.); supawadeethongyim@gmail.com (S.T.); tda007suju@gmail.com (T.K.); tara.chitov@gmail.com (T.C.); sakunnee.b@cmu.ac.th (S.B.); 2 Research Center in Bioresources for Agriculture, Industry and Medicine, Faculty of Science, Chiang Mai University, Chiang Mai 50200, Thailand

**Keywords:** bovine mastitis, endothelial cell, lipopolysaccharide-induced cell death, inflammation, herb extracts, *Clinacanthus nutans* (Burm. f.) Lindau

## Abstract

Mastitis caused by bacterial infection has negative impacts on milk quality and animal health, and ultimately causes economic losses to the dairy industry worldwide. Gram-negative bacteria and their component lipopolysaccharide (LPS) can trigger the inflammatory response of endothelial cells (ECs) and subsequently promote EC dysfunction or injury, which is a critical pathogenesis of mastitis-causing sepsis shock. To control the bacterial infection and to minimise the LPS negative effects on ECs, we thus aimed to identify the potential herb extracts that comprised antibacterial activity and protective ability to inhibit LPS-induced cell death. Extracts from seven types of herbs derived from antibacterial screening were investigated for their protective effects on LPS-stimulated bovine endothelial cell line. *Clinacanthus nutans* (Burm. f.) Lindau (*C. nutans*) extract appeared to be the most effective antiapoptotic extract against LPS stimulation. Treatment of *C. nutans* extract in LPS-stimulated cells significantly lowered apoptotic cell death through modulating pro-survival Bcl-2 and pro-apoptotic Bax expression. The investigation of bioactive compounds using solvent fractionation, HPLC, and LC-MS/MS analysis revealed glyceryl 1,3-disterate (C_39_H_76_O_5_), kaempferol 3-O-feruloyl-sophoroside 7-O-glucoside (C_43_H_48_O_24_), and hydroxypthioceranic acid (C_46_H_92_O_3_) as the candidate components. Our findings indicated that *C. nutans* extract has great potential to be further developed as an alternative therapeutic agent for mastitis treatment.

## 1. Introduction

Mastitis is a prevalent disease in dairy cows that has a negative economic impact on the dairy industry worldwide. The majority of cases of bovine mastitis are caused by bacterial infection of the udder. It is considered one of the most serious diseases as it causes a reduction of milk production during the mammary gland injury, increases veterinary costs, increases the chance of contamination of antibiotic residues in milk, and occasionally causes deaths of dairy cows [1]. Bacteria causing bovine mastitis can be classified into two categories of contagious and environmental pathogens. Contagious pathogens, such as *Staphylococcus aureus*, *Streptococcus agalactiae*, *Mycoplasma* spp., and *Corynebacterium bovis*, can transmit among dairy cows during the lactation period. Environmental pathogens, such as *Escherichia coli*, *Klebsiella* spp., *Streptococcus dysgalactiae*, and *Streptococcus uberis*, can infect dairy cows from the farm environment [2].

Lipopolysaccharide (LPS), a component of all Gram-negative bacteria, contributes to mastitis pathogenesis through promoting an uncontrolled inflammatory response. In many cases, Gram-negative bacteria and their LPS were responsible for acute clinical mastitis, which led to serious complications such as systemic septic shock in dairy cows [3,4]. Endothelial cells (ECs) are one of the first cells to respond to bacterial infection as they are the natural barrier of intravascular components and extravascular tissue [5]. The contributions of EC pathology to the acute phase of bovine mastitis have been demonstrated in several works, which concordantly elucidated the consequence of LPS on stimulating uncontrolled inflammation response and integrity loss of ECs [6].

Until now, there has been accumulated evidence demonstrating the direct effects of LPS on modulating the inflammation and immune response in vitro. Stimulation of cells with LPS strongly increased the production of pro-inflammatory cytokines, that is, interleukin (IL)-6, IL-1β, and IL-8, which likely occurred during sepsis [7,8] in addition to triggered signaling pathway of apoptosis [4,9]. These concrete data not only support the attribution of LPS on EC pathology during mastitis, but also establish a new strategy to ameliorate clinical manifestation of mastitis through attenuation of LPS adverse effects to prevent EC injury. Herb extracts have increasingly received attention because they are considered potential natural alternatives to antibiotics for infectious disease treatment. With a diversity of biological properties, particularly antibacterial and anti-inflammatory properties, as well as being safe according to traditional use, herb extracts and their derivatives are thusthe focus in our study. On the basis of the diverse effects of herb extracts, we hypothesised that herb extracts possibly hold dual functions to inhibit bacterial infection and decrease the adverse effect of the LPS component. In this study, we determined the activity of Thai herb extracts to inhibit LPS-induced cell death. We demonstrated that *C. nutans* ethanolic extract was highly effective by rescuing 95% of bovine endothelial cell line, namely CPAE from LPS stimulation. Therefore, the *C. nutans* extract was further fractionated and characterised for the active fractions. The bioactive compounds of active fractions were further characterised using LC-MS/MS analysis.

## 2. Results

### 2.1. Bovine ECs Highly Sensitive to LPS

**Figure 1:**
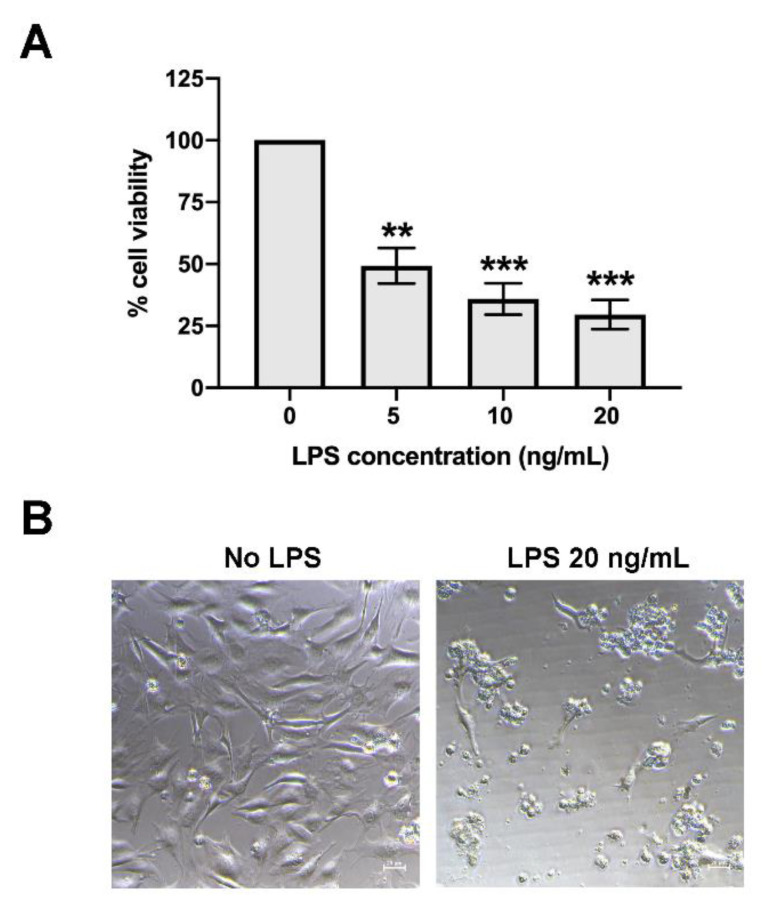
The effect of lipopolysaccharide (LPS) on bovine endothelial cell (EC) death. Bovine ECs were treated with LPS at the concentration of 5–20 ng/mL for 24 h. (**A**) The cell viability was measured using Prestoblue™ reagent, where non-treatment control was set as 100% cell viability (* indicates *p* < 0.05, ** indicates *p* < 0.01, and *** indicates *p* < 0.001). (**B**) The changes in cell morphology upon LPS treatment were observed under the microscope.

Cell death caused by LPS occurs among species; however, bovine ECs showed high sensitivity to the apoptosis-induced effect of LPS [10]. In our study, treatment of low dose LPS (5–20 ng/mL) to LPS-stimulated bovine endothelial cells, CPAE, dramatically induced cell death. The cell viability assay performed using Prestoblue™ reagent revealed that there was approximately 50% cell death upon 24 h treatment with 5 ng/mL LPS (Figure 1A). A dose-dependent effect of LPS was observed, that is, the higher the LPS dose, the higher the percentage of cell death. Treatment with 20 ng/mL LPS (highest dose in this study) caused approximately 70% of cell death (Figure 1A). This was concordant to the dramatic changes in cell morphology observed under the microscope upon LPS treatment compared with that of the control (Figure 1B). Our data supported high sensitivity of bovine EC to LPS.

### 2.2. Anti-Bacteria and Protection Effect of C. Nutans Extract on LPS-Induced EC Cell Death

**Figure 2:**
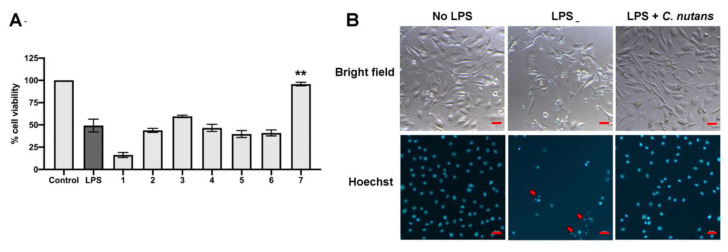
*C. nutans* extract rescuing effect on LPS-induced ECs. In total, seven of the herb extracts including *T. bellirica* (extract 1), *C. sappan* (extract 2), *G. lingzhi* (extract 3), *P. emblica* (extract 4), *K. parviflora* (extract 5), *T. chebula* (extract 6), and *C. nutans* (extract 7) were screened for their activity to protect LPS-induced cell death in ECs. (**A**) Cells were treated with LPS in the presence of herb extracts at the sub-lethal dose for 24 h and the cell viability was measured using Prestoblue™ reagent (* indicates *p* < 0.05, ** indicates *p* < 0.01, and *** indicates *p* < 0.001). (**B**) The changes of cell morphology (upper) and nucleus staining (lower) upon LPS treatment were observed under the microscope. The red arrows indicated the fragments of nucleus. The scaled bar (20 micrometers) was indicated in red.

To identify the novel bioactive compounds for the development of mastitis treatment, Thai herbs are selected for their potential anti-bacterial activity and anti-cell death properties. A total of 14 herbs extracts were selected to test antimicrobial activities based on their traditional use in addition to the reported antimicrobial activities against microorganism (Table 1). Seven herb extracts with strong inhibitory activity on mastitis-causing E. coli were chosen from 14 herb extracts (Table 1). The extracts from *Terminalia bellirica* (extract 1), *Caesalpinia sappan* L. (extract 2), *Ganoderma lingzhi* (extract 3), *Phyllanthus emblica* Linn. (extract 4), *Kaempferia parviflora* (extract 5), *Terminalia chebula* Retz. (extract 6), and *C. nutans* (extract 7) were determined for their ability to protect CPAE cell death after LPS treatment. CPAE was treated with 5 ng/mL LPS for 24 h in the presence or absence of herb extracts. The concentrations of the herb extracts used were those that had been known to be non-toxic to the cells (Supplementary Figure S1). The results showed that treatment with *C. nutans* extract was the most effective. *C. nutans* extract at the concentration of 100 µg/mL significantly rescued CPAE from the LPS damaging effect by approximately 95% (Figure 2A). Furthermore, the microscopic analysis of cell morphology confirmed the strong protective effect of *C. nutans* extract on the endothelial cells (Figure 2B). As nuclear fragmentation is an obvious characteristic of apoptotic cell death, we further investigated the effect of *C. nutans* extract on nuclear fragmentation of LPS-induced cells using Hoechst nuclear staining. Interestingly, treatment of *C. nutans* extract in LPS-treated cells caused a decrease in nuclear fragmentation (Figure 2B), highlighting the potential of the extract in preventing LPS-induced cell death in ECs.

### 2.3. C. Nutans Extract Rescued EC Apoptosis through Modulation of Bcl-2 and Bax Expression

**Figure 3:**
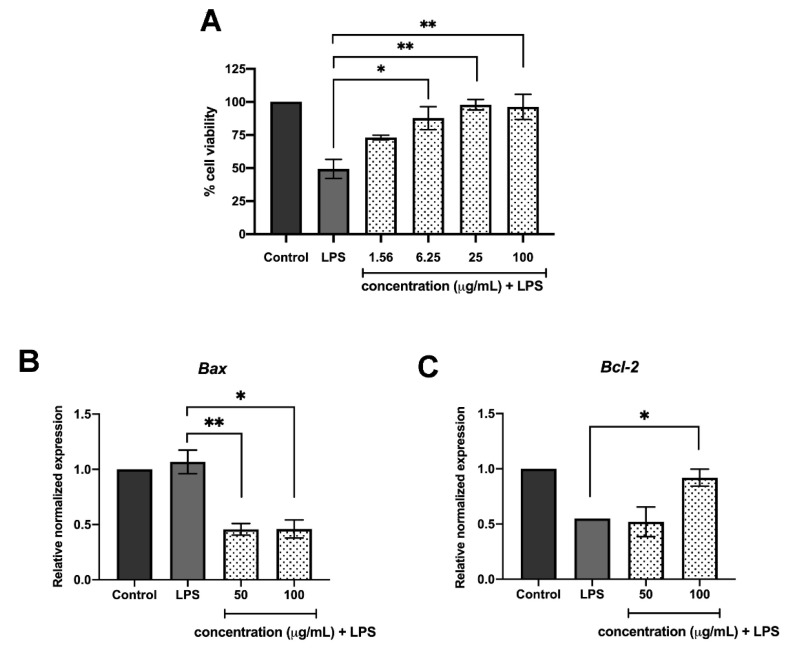
*C. nutans* extract modulation of apoptosis signaling. (**A**) The efficiency of extract to inhibit LPS-induced cell death was tested using varied doses of extracts in the ranges of 1.56–100 µg/mL. (**B**,**C**) The effect of the extract on modulation of the expression of apoptosis related *Bcl-2* and *Bax* gene was determined using qPCR. (* indicates *p* < 0.05, ** indicates *p* < 0.01, and *** indicates *p* < 0.001).

The efficiency and dose-dependent effect of *C. nutans* extract in protecting LPS-induced apoptotic cell death in bovine ECs were characterised. Treatments of LPS-stimulated CPAE with *C. nutans* extracts prepared at various concentrations ranging from 6.25 to 100 µg/mL caused a significant decrease in EC cell death compared with that of the non-treatment control (Figure 3A). To further study its biological mechanism, TUNEL assay was performed to confirm that *C. nutans* extract could protect LPS-induced cell death from apoptosis signaling. The results demonstrated a reduction of positive TUNEL cells in *C. nutans* extract-treated cells (Supplementary Figure S2), which indicated that *C. nutans* extract possibility had an effect on modulating the apoptosis signaling pathway. Accordingly, we studied the effect of the extract treatment on modulating Bcl-2 and Bcl-2-associated X (Bax) gene expression, which plays a vital role in apoptosis signaling induction. Balancing of pro-survival Bcl-2 and pro-apoptosis Bax critically contributed on the regulation of apoptosis. The qPCR results demonstrated that *C. nutans* extract could significantly increase the gene expression of *Bcl-2* but reduce *Bax*. Treatment of LPS induced ECs with 100 µg/mL extract increased *Bcl-2* expression to 0.92 and reduced *Bax* expression 0.45-fold in relation to that of the non-treatment control (Figure 3B,C), thus emphasising the possible mechanism of *C. nutans* extract to manipulate the apoptosis signaling pathway and shield ECs from LPS-induced cell death.

### 2.4. Bioactive Compounds in Active Fractions of C. Nutans Extract

**Figure 4:**
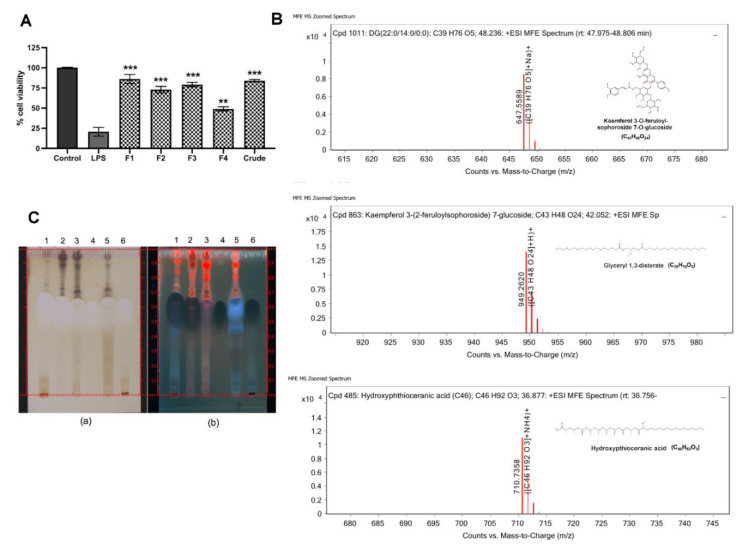
Characterisation of bioactive compounds in *C. nutans* extract. (**A**) The biological activities of four fractions—hexane fraction (F1), dichloromethane fraction (F2), ethyl acetate fraction (F3), and water fraction (F4), respectively—derived from sequential fractionation to reduce LPS-induced cell death were tested using Prestoblue™ reagent (* indicates *p* < 0.05, ** indicates *p* < 0.01, and *** indicates *p* < 0.001). (**B**) The mass spectra and molecular structure of three overlapped majorcompounds—glyceryl 1,3-disterate (C_39_H_76_O_5_), m/z 647.5589, exact mass 624.5681 (B1); kaempferol 3-O-feruloyl-sophoroside 7-O-glucoside (C_43_H_48_O_24_), m/z 949.2620, exact mass 948.2526 (B2); and hydroxypthioceranic acid (C_46_H_92_O_3_), m/z 710.7358, exact mass 692.7030 (B3). (**C**) TLC chromatogram of *C. nutans* extract (1); hexane fraction (2); dichloromethane fraction (3); ethyl acetate fraction (4); glyceryl 1,3-disterate, Rf 0.58 (4); and water fraction (5) using hexane/chloroform/ethyl acetate/methanol/water/formic acids 3:3:2:2:0.1:0.1, sprayed with 0.5% Anisaldehyde-H2SO4 reagent and heat at 120 °C for 10 min, recorded under visible light (a) and UV 366 nm (b).

To identify bioactive compounds from *C. nutans* extract that are responsible for the protection of ECs from LPS-induced cell death, sequential fractionation of *C. nutans* extract was carried out using different solvents including hexane (fraction 1), dichloromethane (fraction 2), ethyl acetate (fraction 3), and water (fraction 4). All fractions at equal concentrations were tested to confirm the biological activities on reducing LPS-induced EC cell death. The results showed that all fractions could reduce the rate of EC cell death after treatment with 10 ng/mL of LPS (Figure 4A). The most effective fraction was the hexane fraction (fraction 1), which could rescue more than 83% of cells from cell death, followed by the ethyl acetate fraction (fraction 3), dichloromethane fraction (fraction 2), and water fraction (fraction 4), which retained approximately 76%, 70%, and 44% of cell viability, respectively (Figure 4A and Supplementary Figure S3). Active fraction 1–3 and less active fraction 4 were analysed for major components using HPLC (Supplementary Figure S4) and LC-MS/MS (Figure 4B). The results revealed that glyceryl 1,3-disterate (C_39_H_76_O_5_), m/z 647.5589, exact mass 624.5681 was the major component in all active fractions as well as in water fraction, which was the less active part (Supplement Data Figures S4–S7). Additionally, kaempferol 3-O-feruloyl-sophoroside 7-O-glucoside (C_43_H_48_O_24_), m/z 949.2620, exact mass 948.2526 was found as the major component, whereas hydroxypthioceranic acid (C_46_H_92_O_3_), m/z 710.7358, exact mass 692.7030 was the minor in the active fractions, but did not present in the water fraction (Supplement Figures S5–S8). These three compounds might represent the candidate active components in the active fractions (Figure 4B). As the glyceryl 1,3-disterate was the only compound commercially available, we were able to confirm the presence of this active compound in all extract fractions using thin layer chromatography (TLC). TLC using hexane/chloroform/ethyl acetate/methanol/water/formic acids (3:3:2:2:0.1:0.1) as mobile phase showed that glyceryl 1,3-disterate (Rf 0.68) was the major component found in *C. nutans* extract and all fractions, both active and inactive (water) parts (Figure 4C). The compound could not be seen under visible light or UV 254 or 366 nm. Therefore, the derivatization of TLC with sprayed reagent had to be performed. After being sprayed with 0.5% Anisaldehyde-H2SO4 sprayed reagent and heat at 120 °C for 10 min, the compound exhibited no color under white light and blue color under UV 366 nm.

## 3. Discussion

Mastitis caused by bacterial infection results in a variety of clinical symptoms in dairy cows including EC injury and death [4,5]. Naturally, the EC layer lining in bovine mammary gland plays an important role as an intact barrier to protect the underlaying tissues from pathogen infection and regulating an optimal response against local infections [5]. The damage of ECs upon LPS exposure consequently breaks down the membrane integrity and promotes the acute inflammation response, which might result in systemic complication of sepsis shock [3]. Usage of broad-protection antibiotics to cure the disease is a common strategy to control bacterial propagation, but this might not be sufficient to rescue EC from the destructive effect of LPS. Therefore, a new approach of treatment that can also neutralise the damaging effect of LPS on ECs is still required to reduce the risk of serious complication occurring during bovine mastitis.

The effects of herb extracts to inhibit LPS-induced cell death were investigated in this study. We selected seven potential herb extracts based on their antibacterial activities against mastitis-causing bacteria to further investigate them for their LPS-neutralising function. As herb extracts are well-known for their broad biological activities, we hypothesised that some herb extracts might have dual functions to inhibit mastitis-causing Gram-negative bacteria and to reduce the effect of their LPS component. On the basis of our experiment results, *C. nutans* extract was the only extract that potentially possessed the dual functions according to our hypothesis. *C. nutans* extract revealed potential effects on protecting more than 95% of cells at the concentration of 100 µg/mL (Figure 2A). The cell viabilities of those treated with the other five herb extracts did not significantly change, except Terminalia bellirica, which dramatically caused cell death. Treatment of *C. nutans* extract was highly effective against cell death and could even significantly reduce EC cell death at the lowest concentration of 6.25 μg/mL (Figure 3A).

LPS triggered the cell death signaling via distinct or overlapping mechanisms [4,9]. The binding of lipid A moiety of LPS and Toll-like receptor 4 (TLR4) activates downstream signaling of nuclear factor-κB (NF-κB) in myeloid differentiation factor 88 (MYD88)-dependent or independent pathway [9,11]. The signaling activation promotes the cascade of caspase function and eventually causes cell apoptosis. Interestingly, the study in murine RAW264.7 macrophage cell line revealed the effect of *C. nutans* extract on the reduction of TLR4 activation, which resulted in the lower production of pro-inflammatory cytokines including tumour necrosis factor (TNF)-α, IFN-γ, IL-1β, IL-6, IL12p40, and IL-17 in LPS-stimulated RAW264.7 cells [12]. It clearly explained the anti-inflammation mechanism of *C. nutans* in response to LPS stimulation. However, no evidence related to the death of RAW264.7 cells was presented, which might be owing to the resistance of RAW264.7 cells to LPS [12]. Besides, the effect of LPS on the regulation of Bcl-2/Bax expression ratio, which is one of the well-known mitochondrial apoptosis mechanisms, has been reported in various cell types in vitro and in vivo [13,14,15,16]. Bax protein plays a role in apoptosis by inducing the release of cytochrome c and other pro-apoptotic factors from the mitochondria resulting in the caspase activation. Recently, the effect of LPS on increasing Bax protein expression was demonstrated in primary bovine mammary cells [16]. Our finding showed the effect of LPS to induce apoptosis based on the appearance of nuclear fragments and TUNEL assay (Figure 2B and Supplementary Figure S2). Treatment of *C. nutans* extract could evidently reduce the degrees of DNA fragmentation (Figure 2B and Figure 3B). As the significant reduction of pro-apoptotic Bax expression was observed in *C. nutans* extract treatment (Figure 3C), we hypothesised that the action of *C. nutans* extract to protect ECs from LPS was through the alteration of Bax expression. However, further investigation is needed to confirm the inhibition of *C. nutans* extract on NF-κB signaling, which is a possible responsible pathway.

*C. nutans* has been used as a traditional medicine in Southeast Asia, including Thailand, Malaysia, and Indonesia, for treating skin rashes, snake and insect bite, dysuria, gout, and diabetes [17,18,19,20]. In Thailand, this plant is in the national list of essential medicines for treatment of herpes simplex and varicella-zoster virus (VZV) infection. According to the potential pharmaceutical use of *C. nutans*, a variety of products is available in the current market in the forms of capsule, tea, tablet, lotion, cream, and so on. For decades, the broad-spectrum pharmacological activities of *C. nutans* have been studied, which revealed anti-inflammation, antioxidant, immune response activity, anti-viral activity, and antivenom activity, which supported the traditional use of this plant. In this present study, the anti-apoptosis activity of *C. nutans* extract against LPS-induced cell death was demonstrated. Phytochemical study to identify the bioactive compounds in *C. nutans* extract was performed using combined solvent fractionation and LC-MS/MS analysis and revealed that glyceryl 1,3-disterate (C_39_H_76_O_5_) was the major component in all extract fractions, which was confirmed by TLC technique. Kaempferol 3-O-feruloyl-sophoroside 7-O-glucoside (C_43_H_48_O_24_) was also the major component and only found in active fractions (fractions 1–3). Although, hydroxypthioceranic acid (C_46_H_92_O_3_) is quite minor, it is found only in active fractions. These compounds might represent the candidate active components of the *C. nutans* extract and active parts. However, there is a possibility that other minor compounds could provide strong biological activities, but be missing owing to the limitation of the technique. Thus, this possibility is not excluded, but requires further investigation.

Previously, various phytochemicals presented in *C. nutans* extract have been reviewed and a range of phytochemical compounds including flavonoids, triterpenoids, steroids, phytosterols, and glycosides have been demonstrated [19,20]. Only a few studies have reported the biological activities of the compounds identified as potential bioactive compounds in this study, and most of them were related to anti-inflammatory and antioxidant activities. Yu and colleagues demonstrated the anti-inflammatory and anti-obesity of glyceryl 1,3-disterate (C_39_H_76_O_5_) containing medium-chain fatty acid-diacylglycerols (MCDGs) [21]. Interestingly, the treatment of MCDG could potentially reduce the inflammation response in LPS-stimulated macrophage by downregulating the expression of cyclooxygenase-2 (COX-2), inducible nitric oxide synthase (iNOS), and inflammatory cytokines—interleukin-6 (IL6) and tumour necrosis factor alpha (TNF alpha) [21]. The inflammation response, particularly the increase of TNF alpha, is able to manipulate caspase activation, a reactive oxygen species (ROS) product that ultimately causes cell apoptosis [22,23]. Thus, glyceryl 1,3-disterate, which decreases the expression of TNF alpha, probably rescues ECs to prevent the signaling of apoptosis.

A wide range of pharmacological activities of dietary flavonoid kaempferol and glycosides of kaempferol has been reported including antioxidant, anti-inflammatory, antimicrobial, antidiabetic, and neuroprotective [24,25]. Concordant results have been shown in several works that demonstrated the effect of kaempferol on modulating antiinflammation response via inhibiting NF-kB activity [24]. Furthermore, treatment of kaempferol in P12 rat pheochromocytoma cell line could strongly diminish the ROS production in response to H_2_O_2_ and significantly increase the cell viability, which suggested the role of kaempferol in inhibiting apoptosis [26]. Although further investigation is needed, according to the literature, the bioactive compounds of *C. nutans* extract that confer its protective effect on EC cell death probably included at least the glyceryl 1,3-disterate and kaempferol.

Currently, the acquired resistance to antibiotic drugs used for treatment of bovine mastitis has been reported globally. This fact has raised serious concern and might limit the antibiotic utility in the future. Accordingly, there were many attempts to identify potential herbs or medicinal plants owing to their broad biological activities and their safety to replace antibiotic usage. The increasing publications demonstrating the effects of herb extracts or natural compounds to prevent inflammation and damage of ECs during the past few years clearly emphasise the importance of EC pathogenesis during mastitis [16,27,28]. On the basis of our findings in this study, *C. nutans* extract, which had both antibacterial and anti-apoptosis activities, could be an alternative treatment for mastitis to simultaneously regulate the spread or infection of bacteria and to reduce the adverse effect of LPS. Owing to the safety of this plant, the extract can be applied as intramammary infusion to treat local infection or as dietary product to control systemic infection. A field study based on these laboratory results is still required to confirm the safety and to evaluate the efficacy of *C. nutans* extract to reduce the degree of LPS-induced cell death in vivo, which, if successful, could provide a basis for a novel approach for bovine mastitis therapy in the future.

## 4. Materials and Methods 

### 4.1. Herb Extraction

Thai herbs including *C. nutans* (from leaves), *Terminalia bellirica* (from fruit), *Caesalpinia sappan* L. (from wood), *Kaempferia parviflora* (from rhizome), *Phyllanthus emblica* Linn. (from fruit), *Ganoderma lingzhi* (from fruiting body), and *Terminalia chebula* Retz. (from fruit) were collected from Chiang Mai, Thailand during September–October 2010. All specimens were identified by Dr. Narin Printarakul, taxonomist, Department of Biology, Faculty of Science, Chiang Mai University. They were cleaned with tap water and dried in a hot air oven at 60 °C for 24 h. The 1 kg of dried samples was ground using electronic blender into powder followed by extraction by maceration with 70% EtOH at 1:20 *v*/*v*. The mixture was shaken at 160 rpm at room temperature for 12 h and then filtered through Whatman^®^ filter paper no. 1. The filtrate was concentrated using a rotary evaporator, dried on a water bath, and kept in a sealed brown glass bottle at 4 °C in a refrigerator until use.

### 4.2. Fractionation of Herb Extracts 

*C. nutans* extract was sequentially fractionated by hexane, dichloromethane, ethyl acetate, and 5% methanol (MeOH)/water *v*/*v*. Firstly, 15 g of the extract was dissolved in 315 mL of 5% MeOH/water using sonication. The MeOH part was partitioned with hexane using a separatory flask at a 1:1 (*v*/*v*) ratio three times. The hexane part was separated, and the methanol part was further partitioned followed by dichloromethane and ethyl acetate at 1:1 (*v*/*v*) ratio, respectively. All fractions were evaporated using a rotary evaporator, dried in a laboratory fume hood, and kept in a sealed brown glass bottle at 4 °C in a refrigerator until use.

### 4.3. Agar Disc Diffusion Assay

The antimicrobial activity of the herbs extract was determined by agar disc diffusion assay. All herbs were dissolved in dimethyl sulfoxide (DMSO) at a concentration of maximum solubility of 500 mg/mL. *E. coli* Department of Medical Science Thailand 703 (DMST 703) was obtained from the Department of Medical Sciences, Ministry of Public Health, Thailand. Briefly, *E. coli* was cultivated in Mueller–Hinton (MH) broth (Difco™, MD, NJ, USA) and incubated at 37 °C for 18–24 h. Turbidity of the bacterial culture was adjusted to McFarland standards No. 0.5, which corresponded to 1.5 × 10^8^ CFU/mL. The culture of bacteria was then swabbed on Mueller–Hinton (MH) agar (Difco™, MD, NJ, USA). Afterwards, paper discs (Macherey-Nagel, Duren, Germany) were soaked in 500 mg/mL of each extract and placed on the agar. The antibiotic gentamycin was used as a positive control of the experiment. The highest concentration of 500 mg/mL extract and 1 mg/mL of gentamycin were used in this study in order to determine the maximum anti-bacterial activity and effective diffusion of the extracts released from agar disc to inhibit the bacteria. The plates were incubated at 37 °C for 18–24 h. Finally, the antibacterial activity was determined by measuring the zone of growth inhibition.

### 4.4. Cell Lines and Reagents

Bovine endothelial cell line, CPAE (CCL209TM), was purchased from American Type Culture Collection (ATCC, VA, USA) and cultured in minimal essential medium (MEM) supplemented with 20% (*v*/*v*) fetal bovine serum (FBS) and antibiotics at 37 °C under 5% CO2.

### 4.5. Cell Viability and TUNEL Staining Assay

To determine the viability of the CPAE endothelial cells after LPS treatment, CPAE was plated the day prior to the experiment at a density of approximately 7000 cells/well in the 96-well format microtiter plates. At the time of the experiment, LPS was prepared at the tested concentration of 5, 10, and 20 ng/mL in culture media (LPS stock concentration = 100 μg/mL) and added to the monolayer of the cells. The screening assay to determine the effect of herb extract to prevent cell death, and the final concentration of 5 ng/mL LPS was used and added to the cells in the presence or absence of herb extract at the concentration that was not toxic to the cells. The used volumes of all herb extracts were less than 0.1% *v*/*v*. Treated cells were harvested at 24 h after incubation and the cell viability determined using prestoBLUE^TM^ cell viability reagent (Invitrogen, MA, USA) according to the manufacturer’s protocol. The absorbance was monitored to detect the changes in reagent colors, which were related to the reduced ability of living cells, at OD570 and OD595. The percentage of cell viability was calculated and compared to that of non-treated cells (set as 100% cell viability), as follows: 

percentage of cell viability = [(OD570-OD595) treated cells/(OD570-OD595) non-treated cells] ×100.

### 4.6. TUNEL Assay

Detection of the late apoptosis of CPAE cells induced with LPS after treatment with *C. nutans* extract was investigated using TUNEL assay, using the DNA Fragmentation Imaging kit (Merck, Darmstadt, Germany). CPAE cells induced with LPS were treated with *C. nutans* extract (25, 50, and 100 µg/mL) for 24 h. After incubation, the cells were washed with phosphate buffer saline (PBS, pH 7.4) three times. The cells were then fixed with 100 µL of 4% paraformaldehyde and incubated at room temperature for 10 min. One hundred microlitres of 0.1% Triton-X100 was added after removing the fixing solution and the cells were then incubated at room temperature for 20 min. After washing twice with PBS, the solution of terminal deoxynucleotidyl transferase (TdT) enzyme was added and the cells were incubated at 37 °C for 1 h. Then, nuclei dye mixture solution (Hoechst) was added and the cells were further incubated in the dark at room temperature for 15 min. The solution was removed, and the cells were mounted with ProLongTM gold antifade mountant (Life technologies, Camarillo, CA, USA) before detection using an inverted fluorescence microscope (ECLIPSE Ts2R-FL, Nikon, Tokyo, Japan).

### 4.7. Quantitative Reverse Transcription Real-Time PCR (qRT-PCR)

To investigate the effect of *C. nutans* extract on gene expression of BAX, qRT-PCR was carried out. Briefly, CPAE was plated the day prior to the experiment at 50,000 cells/ well (in the six-well format microtiter plate). At the time of the experiment, LPS (10 ng/mL) was added to the cells in the presence of *C. nutans* extract at concentrations of 25, 50, and 100 µg/mL. At 6 h of incubation, the treated cells were harvested. Total RNA was extracted, and a 500-ng portion of RNA was converted into cDNA using TriZol reagent (Invitrogen, Carlsbad, CA, USA) and cDNA synthesis kit (Toyobo Life Science, Osaka, Japan). Real-time PCR was performed using SensiFAST™ SYBR® (Bioline, London, UK) and PCR primers as follows: Bax: 5′ATGACTTCTCTCGGCGCT3′ (forward) and 5′CGGTTCAGGTACTCGGTCAT3′ (reverse); GAPDH: 5′GCTGCCCAGAATATCATCCCT3′ (forward) and 5′GCAGGTCAGATCCACAACAG3′ (reverse). The sample analysis was carried out through Bioer Fluorescent Quantitative PCR Detection System, model FQD-96A (Hangzhou Bioer Technology, Zhejiange, China). Achieved cycle thresholds (Ct) were analysed with the ΔΔCT method to determine the differences in the expression. Briefly, the Ct values of samples were normalized to housekeeping gene (*GAPDH*) and relative to the expression of non-treatment control (set as 1). The analysed data from three independent experiments were tested for statistical differences using Student’s *t*-test.

### 4.8. Qualitative Analysis of C. Nutans Extract Using LC-MS/MS

HPLC-UV/DAD of four separate *C. nutans* extracts was performed using Agilent 1260 Infinity DAD detector (Santa Clara, CA, USA) with Agilent Zorbax Eclipse XDB - C18 column (5 µm, 4.6 × 150 mm). The extract was dissolved in methanol (HPLC grade) at a concentration of 5 mg/mL. The extract was analyzed. The 10 µl of extract solution was injected using 0.5% glacial acetic acid (A) and methanol (A) as mobile phase with gradient system, flow rate 1 mL/min, for 60 min. The gradient program was 100% A (0–30 min), 50% A (30–40 min), 100% B (40–50 min), 100% A (50–60 min). This was followed by a 10 min equilibrium period prior to the injection of the next sample. Signal was monitored at 254 nm and 280 nm.

Four *C. nutans* extract fractions were dissolved in DMSO in the concentration of 5000 ppm for further analysis with LC-MS/MS (Agilent 6545 LC/Q-TOF, Agilent, CA, USA) using Porshell 120 EC-C18, 2.7 µm, 2.1 × 100 mm C18 column, with gradient system of two mobile phases (A) water and 0.1% acetic acid and (B) acetonitrile (CAN) and 0.1% acetic acid for 60 min. The gradient elution program was 100% A (0–30 min), 50% A (30–40 min), 0% A (40–51 min), 50% A (51–60 min). This was followed by a 10 min equilibrium period prior to the injection of the next sample. The injection volume of each sample was 3 µl, flow rate 0.2 mL/min. The UV spectra were recorded between 210 and 400 nm. Conditions for MS analysis of each HPLC peak included MS positive mode, Dual AJS ESI ion source, 320 °C gas temperature, 8 L/min drying gas, 35 psig nebulizer, 350 °C sheath gas temperature, 11 L/min sheath gas flow.

Thin layer chromatography (TLC) of *C. nutans* extract and its fractions compared with glyceryl 1,3-disterate (Sigma-Aldrich, St. Louis, MO, USA) was performed using hexane/chloroform/ethyl acetate/methanol/water/formic acids (3:3:2:2:0.1:0.1) as mobile phase. Then, it was derivatized with 0.5% Anisaldehyde-H2SO4 reagent, and then heated on a hot plate at 120 °C for 10 min. The chromatogram was documented under visible light and UV 366 nm using CAMAG Linomat 5 (Camag Chemie-Erzeugnisse und Adsorptionstechnik AG, Muttenz, Switzerland). The retention time (Rf) was recorded.

### 4.9. Statistical Analysis

Bar graphs representing mean and standard error of the mean (SEM) were plotted from at least three independent measurements. The statistical analyses were performed using Student’s *t*-test of GraphPad Prism Software version 8 (GraphPad Software, Inc., La Jolla, CA, USA), where statistical differences were indicated as follows: * indicates *p* < 0.05, ** indicates *p* < 0.01, and *** indicates *p* < 0.001.

## Figures and Tables

**Table 1:** Screening of antibacterial activity of herb extracted. In total, 14 herbs were extracted using ethanol/water at 7:3. Antibacterial activity of herb extracts against *E. coli* was tested at the equal concentrations of extracts (500 mg/mL) using the disc diffusion agar agar method. The clear zone was measured and summarized in the table (mean ± standard deviation) In total, 14 herbs were extracted using ethanol/water at 7:3. Antibacterial activity of herb extracts against *E. coli* was tested at the equal concentrations of extracts (500 mg/mL) using the disc diffusion agar agar method. The clear zone was measured and summarized in the table (mean ± standard deviation).

Herb Extracts	Family Names	Clear Zone (mm)
*Terminalia bellirica*	Combretaceae	11.67 ± 0.58
*Caesalpinia sappan* L.	Fabaceae	11.50 ± 0.71
*Ganoderma lingzhi*	Ganodermataceae	10.33 ± 0.58
*Phyllanthus emblica* Linn.	Phyllanthaceae	10.00 ± 0
*Kaempferia parviflora*	Zingiberaceae	9.00 ± 0.00
*Terminalia chebula* Retz.	Combretaceae	8.00 ± 0.71
*Clinacanthus nutans* (Burm. f.) Lindau	Acanthaceae	7 ± 0.00
*Momordica charantia*	Cucurbitaceae	0
*Andrographis paniculata*	Acanthaceae	0
*Zingiber officinale*	Zingiberaceae	0
*Garcinia mangostana*	Clusiaceae	0
*Curcuma longa*	Zingiberaceae	0
*Alpinia galanga*	Zingiberaceae	0
*Zingiber montanum*	Zingiberaceae	0
Gentamycin (1 mg/mL)		20.50 ± 0.71

## Supplementary Materials

The following are available online at https://www.mdpi.com/2079-6382/9/7/429/s1, Figure S1: Cytotoxicity of *C. nutans* extract fractions; Figure S2: Antiapoptotic activity of *C. nutans* by TUNEL assay; Figure S3 Antiapoptotic activities of *C. nutans* fractions; Figure S4 HPLC profile of *C. nutans* fractions; Figures S5–S8 LC-MS/MS data result of *C. nutans* fractions.
