# Supplementary material for: Antibiotic-Antiapoptotic Dual Function of Clinacanthus nutans (Burm. f.) Lindau Leaf Extracts against Bovine Mastitis
Source: Antibiotics (Basel). 2020 Jul 21;9(7):429. doi: 10.3390/antibiotics9070429 (PMC7400556; doi:10.3390/antibiotics9070429)
Supplement: Supplementary file 1 [file antibiotics-09-00429-s001.pdf]

Supplementary Figures

Figure S1 Cytotoxicity of *C.nutans* extract fractions The various concentrations of the herb extracts were tested for cytotoxicity to CPAE cells using prestoBLUE^{TM} cell viability reagent (Invitrogen, MA, USA). The viability at 24 hours after treatment was calculated relative to that of non-treatment control.

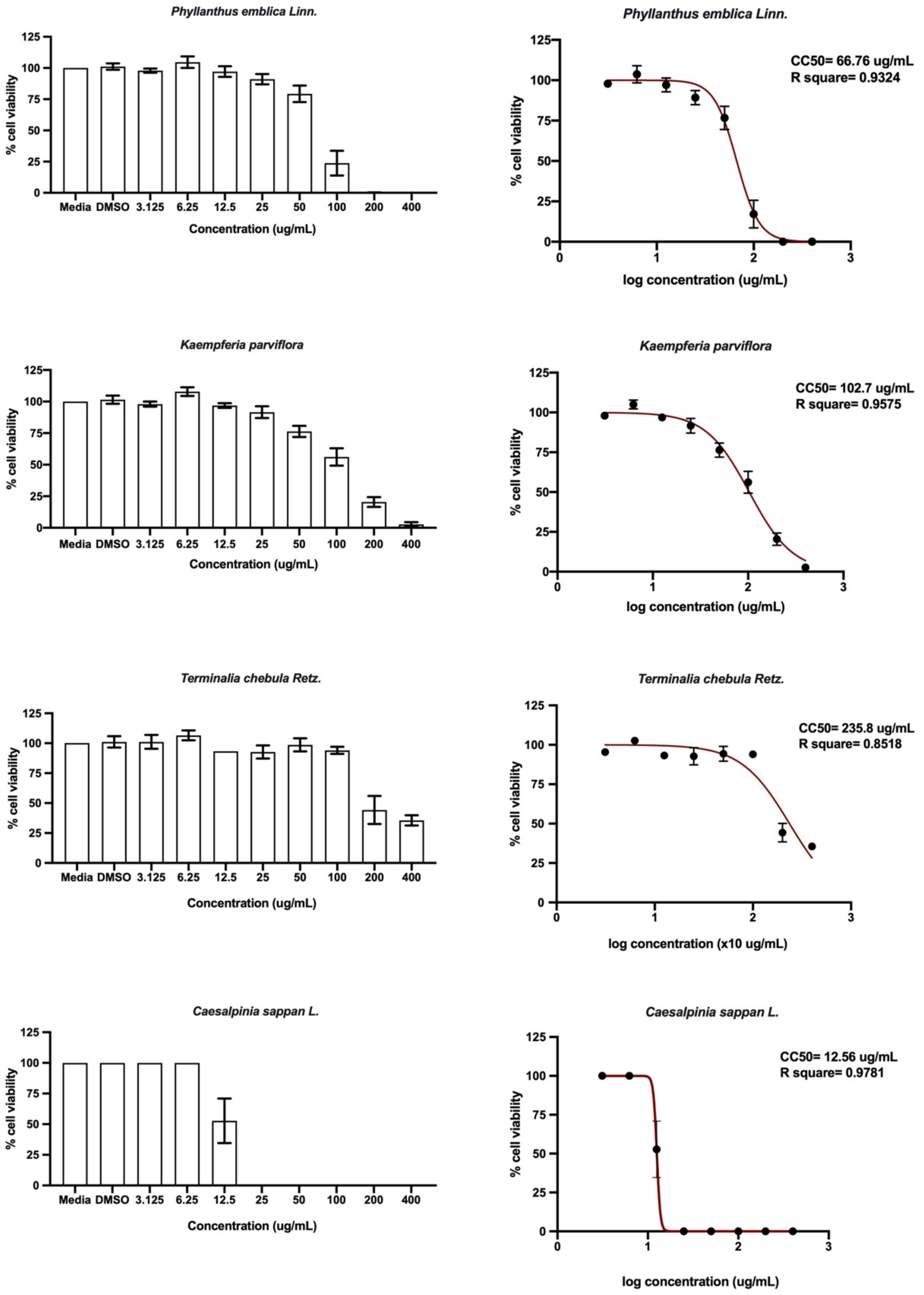

Figure S1 (Continue) Cytotoxicity of *C.nutans* extract fractions The various concentrations of the herb extracts were tested for cytotoxicity to CPAE cells using prestoBLUE^{TM} cell viability reagent (Invitrogen, MA, USA). The viability at 24 hours after treatment was calculated relative to that of non-treatment control.

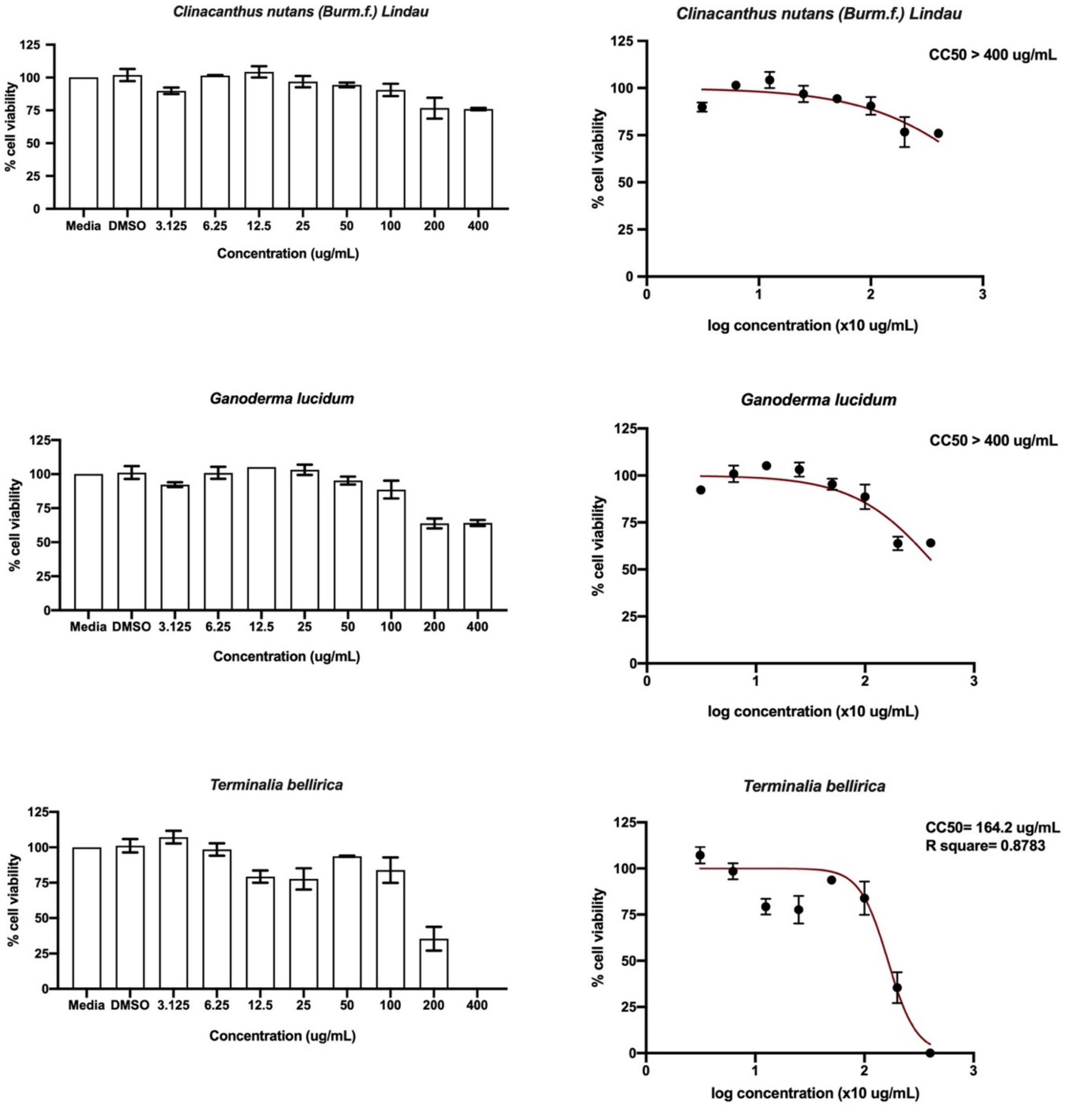

Figure S2 Antiapoptotic activity of *C. nutans* by TUNEL assay. The mode of action of the extract to manipulate apoptosis in LPS-treated cells was confirmed by staining fragmented DNA by TUNEL assay.

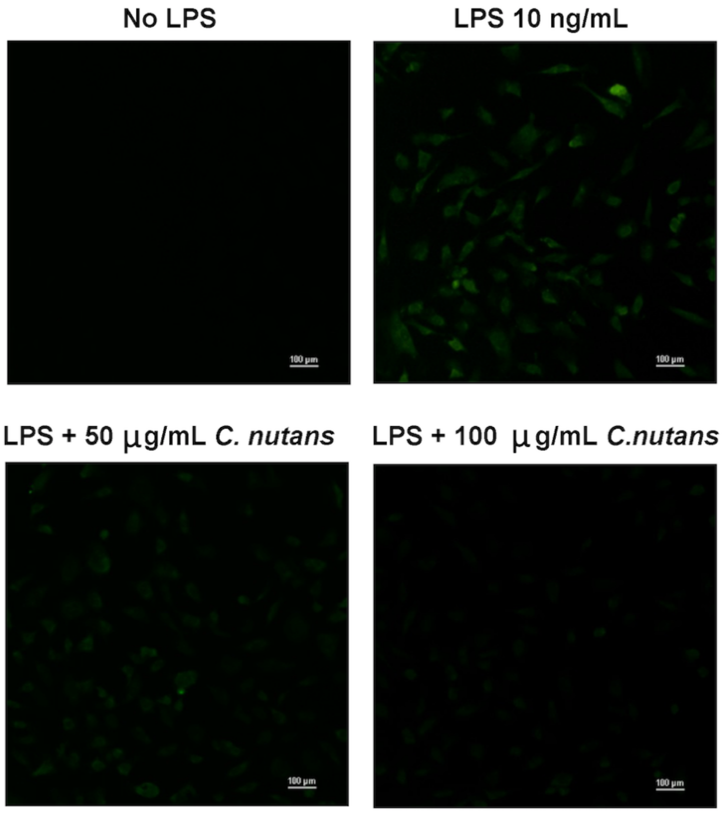

Figure S3 Antiapoptotic activities of C. nutans fractions The changes in cell morphology upon LPS treatment in presence or absence of extract fractions were observed under the microscope.

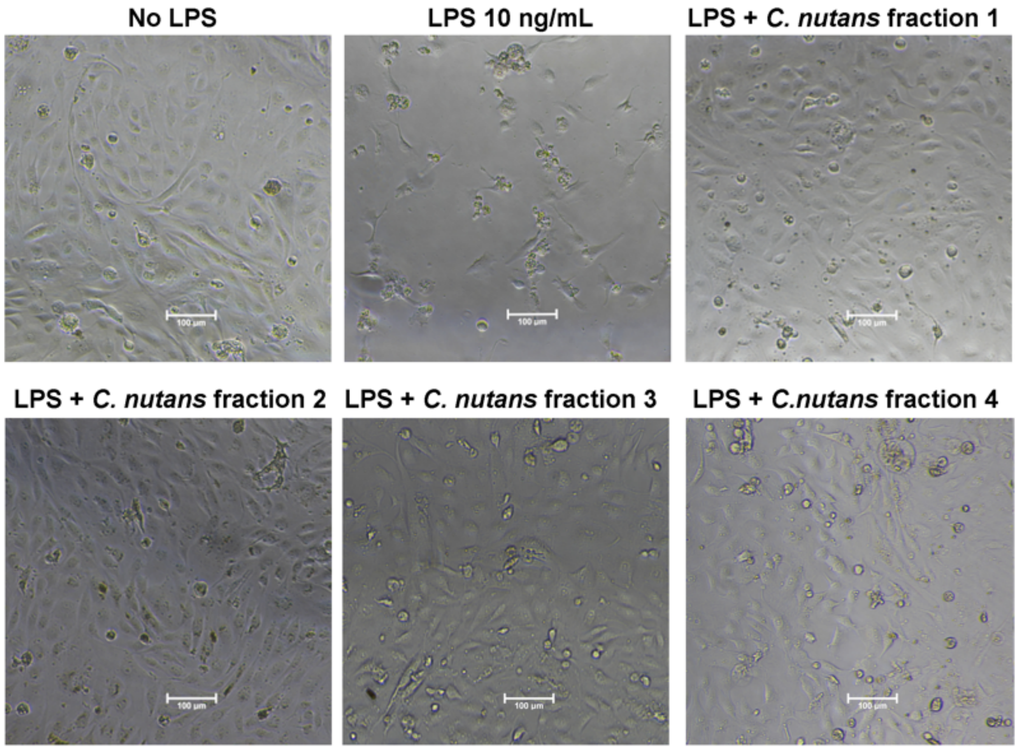

Figure S4 HPLC profile *C.nutrans* extract fractions including hexane (fraction 1), dichloromethane (fraction 2), ethyl acetate (fraction 3), and water (fraction 4) were analysed using HPLC

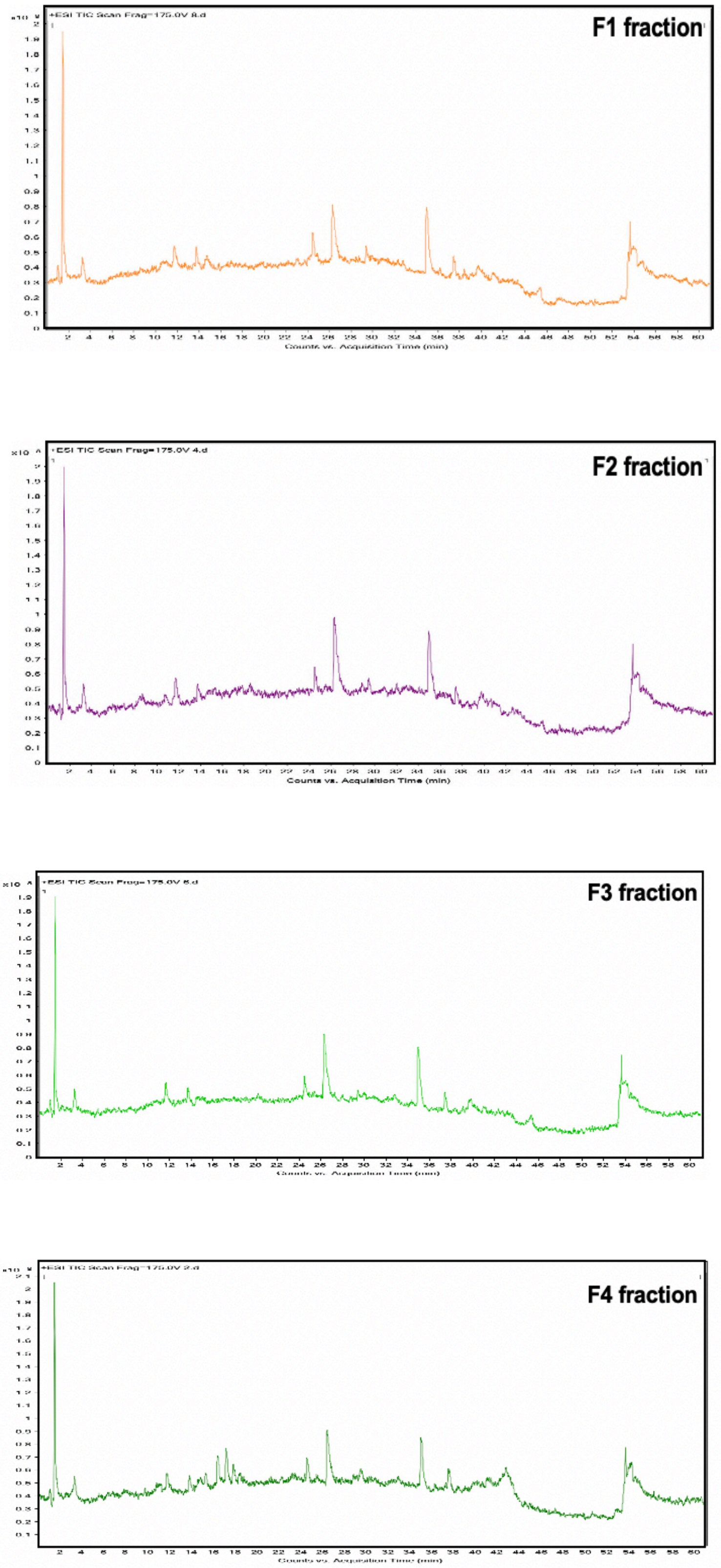

Figure S5 LC-MS/MS analysis of fraction 1 LC-MS/MS data result of hexane fraction (fraction 1) presented three highlighted overlapped components; glyceryl 1,3-disterate ($C_{39}H_{76}O_5$), kaempferol 3-O-feruloyl-sophoroside 7-O-glucoside ($C_{43}H_{48}O_{24}$ and hydroxypthioceranic acid ($C_{46}H_{92}O_3$).

Compound Table

Compound Label	RT	Mass	Name	DB Formula	DB Diff (ppm)	Hits (DB)
Cpd 3: Perindoprilat; C17 H28 N2 O5; 1.522	1.522	340.2002	Perindoprilat	C17 H28 N2 O5	-1.13	1
Cpd 7: Furo[3,4-b]pyridine-3-carboxylic acid, 5,7-dihydro-2-methyl-4-(2-nitrophenyl)-5-oxo-, methyl ester; C16 H12 N2 O6; 10.738	10.738	328.0698	Furo[3,4-b]pyridine-3-carboxylic acid, 5,7-dihydro-2-methyl-4-(2-nitrophenyl)-5-oxo-, methyl ester	C16 H12 N2 O6	-0.69	1
Cpd 51: Chrysoeriol 7-O-(6"-malonyl-glucoside); C25 H24 O14; 14.522	14.522	548.1157	Chrysoeriol 7-O-(6"-malonyl-glucoside)	C25 H24 O14	1.58	8
Cpd 60: Debromoaplysiatoxin; C32 H48 O10; 15.001	15.001	592.3234	Debromoaplysiatoxin	C32 H48 O10	2.29	1
Cpd 66: Neoliquiritin 2"-apioside; C26 H30 O13; 15.239	15.239	550.1677	Neoliquiritin 2"-apioside	C26 H30 O13	1.64	8
Cpd 260: Yuccaol C; C30 H22 O10; 26.555	26.555	542.1212	Yuccaol C	C30 H22 O10	0.18	3
Cpd 261: Isoneotheaflavin; C29 H24 O12; 26.556	26.556	564.1266	Isoneotheaflavin	C29 H24 O12	0.38	6
Cpd 360: PG(O-18:0/22:0); C46 H93 O9 P; 32.307	32.307	820.6564	PG(O-18:0/22:0)	C46 H93 O9 P	-0.89	10
Cpd 361: ergosta-3beta,5alpha,6beta,25-tetrol; C28 H50 O4; 32.314	32.314	450.3718	ergosta-3beta,5alpha,6beta,25-tetrol	C28 H50 O4	-2.01	2
Cpd 467: 5alpha-Ethoxy-6beta-hydroxy-5,6-dihydrophysalin B; C30 H36 O11; 36.047	36.047	572.2247	5alpha-Ethoxy-6beta-hydroxy-5,6-dihydrophysalin B	C30 H36 O11	1.92	1
Cpd 516: Mebendazole; C16 H13 N3 O3; 36.777	36.777	295.0968	Mebendazole	C16 H13 N3 O3	-3.8	1
Cpd 520: Hydroxyphthioceranic acid (C46); C46 H92 O3; 36.825	36.825	692.7054	Hydroxyphthioceranic acid (C46)	C46 H92 O3	-1.14	1
Cpd 524: DG(18:1(11Z)/22:4(7Z,10Z,13Z,16Z)/0:0); C43 H74 O5; 36.922	36.922	670.5546	DG(18:1(11Z)/22:4(7Z,10Z,13Z,16Z)/0:0)	C43 H74 O5	-1.52	10
Cpd 556: 3'-Deoxyoleacein; C17 H20 O5; 37.249	37.249	304.1321	3'-Deoxyoleacein	C17 H20 O5	-3.39	5
Cpd 581: Nocardicin F; C19 H17 N3 O7; 37.440	37.44	399.1067	Nocardicin F	C19 H17 N3 O7	-0.2	2
Cpd 649: 6-Geranylgeranyl 8'-methyl 6,8'-diapocarotene-6,8'-dioate; C43 H60 O4; 38.702	38.702	640.4485	6-Geranylgeranyl 8'-methyl 6,8'-diapocarotene-6,8'-dioate	C43 H60 O4	1.01	1
Cpd 717: (23S)-23,25-dihdroxy-24-oxovitamine D3 23-(beta-glucuronide); C33 H50 O10; 39.653	39.653	606.3399	(23S)-23,25-dihdroxy-24-oxovitamine D3 23-(beta-glucuronide)	C33 H50 O10	0.85	3
Cpd 758: Lys Pro Trp; C22 H31 N5 O4; 40.089	40.089	429.2389	Lys Pro Trp	C22 H31 N5 O4	-2.98	6
Cpd 827: Cyclohex-1,4-diene-1-carboxyl-CoA; C28 H42 N7 O17 P3 S; 41.199	41.199	873.1565	Cyclohex-1,4-diene-1-carboxyl-CoA	C28 H42 N7 O17 P3 S	0.66	3
Cpd 863: Kaempferol 3-(2-feruloylsophoroside) 7-glucoside; C43 H48 O24; 42.052	42.052	948.255	Kaempferol 3-(2-feruloylsophoroside) 7-glucoside	C43 H48 O24	-1.57	10
Cpd 880: PG(17:1(9Z)/12:0); C35 H67 O10 P; 42.356	42.356	678.4481	PG(17:1(9Z)/12:0)	C35 H67 O10 P	-1.41	9
Cpd 890: Ganoderic acid Mc; C36 H54 O9; 42.649	42.649	630.3797	Ganoderic acid Mc	C36 H54 O9	-4.63	4
Cpd 962: Ephedrannin A; C30 H20 O11; 44.407	44.407	556.0995	Ephedrannin A	C30 H20 O11	1.82	1
Cpd 1011: DG(22:0/14:0/0:0); C39 H76 O5; 48.236	48.236	624.5704	DG(22:0/14:0/0:0)	C39 H76 O5	-1.88	10

Figure S6 LC-MS/MS analysis of fraction 2 LC-MS/MS data result of dichloromethane fraction (fraction 2) presented three highlighted overlapped components; glyceryl 1,3-disterate ($C_{39}H_{76}O_5$), kaempferol 3-O-feruloyl-sophoroside 7-O-glucoside ($C_{43}H_{48}O_{24}$ and hydroxypthioceranic acid ($C_{46}H_{92}O_3$).

Compound Table

Compound Label	RT	Mass	Name	DB Formula	DB Diff (ppm)	Hits (DB)
Cpd 1: V-PYRRO/NO; C6 H11 N3 O2; 1.024	1.024	157.0849	V-PYRRO/NO	C6 H11 N3 O2	1.74	6
Cpd 30: Glucosyl (2E,6E,10x)-10,11-dihydroxy-2,6-farnesadienoate; C21 H36 O9; 12.743	12.743	432.2375	Glucosyl (2E,6E,10x)-10,11-dihydroxy-2,6-farnesadienoate	C21 H36 O9	-3.58	1
Cpd 63: Amitrole; C2 H4 N4; 14.673	14.673	84.0439	Amitrole	C2 H4 N4	-3.5	1
Cpd 68: Debromoaplysiatoxin; C32 H48 O10; 14.967	14.967	592.3258	Debromoaplysiatoxin	C32 H48 O10	-1.71	2
Cpd 79: Neoliquiritin 2"-apioside; C26 H30 O13; 15.206	15.206	550.1681	Neoliquiritin 2"-apioside	C26 H30 O13	1	8
Cpd 277: Eprosartan; C23 H24 N2 O4 S; 21.461	21.461	424.1463	Eprosartan	C23 H24 N2 O4 S	-1.51	1
Cpd 286: Hispidulin 7-(6''-E-p-Coumaroylglucoside); C31 H28 O13; 22.182	22.182	608.1547	Hispidulin 7-(6''-E-p-Coumaroylglucoside)	C31 H28 O13	-2.83	4
Cpd 297: Hispidulin 7-(6''-E-p-Coumaroylglucoside); C31 H28 O13; 23.049	23.049	608.1536	Hispidulin 7-(6''-E-p-Coumaroylglucoside)	C31 H28 O13	-0.96	4
Cpd 299: Coroloside; C35 H54 O12; 23.208	23.208	666.3617	Coroloside	C35 H54 O12	-0.28	1
Cpd 349: Albafuran C; C34 H28 O9; 26.551	26.551	580.1742	Albafuran C	C34 H28 O9	-1.58	2
Cpd 351: Yuccaol C; C30 H22 O10; 26.561	26.561	542.1224	Yuccaol C	C30 H22 O10	-2.1	3
Cpd 352: Isoneotheaflavin; C29 H24 O12; 26.563	26.563	564.1278	Isoneotheaflavin	C29 H24 O12	-1.81	4
Cpd 477: Huratoxin; C34 H48 O8; 32.613	32.613	584.3364	Huratoxin	C34 H48 O8	-2.45	4
Cpd 482: Gancaonin H; C25 H24 O6; 32.794	32.794	420.1579	Gancaonin H	C25 H24 O6	-1.56	10
Cpd 504: Armillane; C23 H32 O7; 33.774	33.774	420.2159	Armillane	C23 H32 O7	-2.71	1
Cpd 558: N-cis-Feruloyltyramine; C18 H19 N O4; 35.790	35.79	313.1319	N-cis-Feruloyltyramine	C18 H19 N O4	-1.7	10
Cpd 620: Tyrosyl-Asparagine; C13 H17 N3 O5; 36.745	36.745	295.1173	Tyrosyl-Asparagine	C13 H17 N3 O5	-1.58	7
Cpd 624: Hydroxyphthioceranic acid (C46); C46 H92 O3; 36.819	36.819	692.7026	Hydroxyphthioceranic acid (C46)	C46 H92 O3	2.98	1
Cpd 630: DG(18:1(11Z)/20:1(11Z)/0:0); C41 H76 O5; 36.919	36.919	648.5703	DG(18:1(11Z)/20:1(11Z)/0:0)	C41 H76 O5	-1.58	10
Cpd 636: 3-Epipapyriferic acid; C35 H56 O8; 37.014	37.014	604.3994	3-Epipapyriferic acid	C35 H56 O8	-3.1	3
Cpd 637: Ginsenoside Rh4; C36 H60 O8; 37.028	37.028	620.4292	Ginsenoside Rh4	C36 H60 O8	-0.59	4
Cpd 661: TG(12:0/13:0/14:1(9Z))[iso6]; C42 H78 O6; 37.225	37.225	678.581	TG(12:0/13:0/14:1(9Z))[iso6]	C42 H78 O6	-1.69	2
Cpd 669: 1α,25-dihydroxy-26,27-dimethylvitamin D3 / 1α,25-dihydroxy-26,27-dimethylcholecalciferol; C29 H48 O3; 37.380	37.38	444.3608	1α,25-dihydroxy-26,27-dimethylvitamin D3 / 1α,25-dihydroxy-26,27-dimethylcholecalciferol	C29 H48 O3	-1.06	10
Cpd 750: Dihydromorelloflavone; C30 H22 O11; 38.409	38.409	558.1169	Dihydromorelloflavone	C30 H22 O11	-1.17	2
Cpd 768: Yuccaol C; C30 H22 O10; 38.627	38.627	542.1209	Yuccaol C	C30 H22 O10	0.78	3
Cpd 772: PA(P-18:0/13:0); C34 H67 O7 P; 38.691	38.691	618.4639	PA(P-18:0/13:0)	C34 H67 O7 P	-2.42	3
Cpd 789: UDP-N-acetyl-6-(D-galactose-1-phospho)-D-glucosamine; C23 H38 N3 O25 P3; 38.977	38.977	849.1006	UDP-N-acetyl-6-(D-galactose-1-phospho)-D-glucosamine	C23 H38 N3 O25 P3	0.16	1
Cpd 836: Huratoxin; C34 H48 O8; 39.651	39.651	584.3365	Huratoxin	C34 H48 O8	-2.62	3
Cpd 869: Abscisic alcohol 11-glucoside; C21 H32 O8; 40.082	40.082	412.2105	Abscisic alcohol 11-glucoside	C21 H32 O8	-1.98	2
Cpd 927: Cyclohex-1,4-diene-1-carboxyl-CoA; C28 H42 N7 O17 P3 S; 41.205	41.205	873.1573	Cyclohex-1,4-diene-1-carboxyl-CoA	C28 H42 N7 O17 P3 S	-0.3	3
Cpd 955: Kaempferol 3-(2-feruloylsophoroside) 7-glucoside; C43 H48 O24; 42.034	42.034	948.2513	Kaempferol 3-(2-feruloylsophoroside) 7-glucoside	C43 H48 O24	2.33	10
Cpd 956: PA(18:0/14:1(9Z)); C35 H67 O8 P; 42.076	42.076	646.4576	PA(18:0/14:1(9Z))	C35 H67 O8 P	-0.33	10
Cpd 969: Dapiprazole; C19 H27 N5; 42.386	42.386	325.227	Dapiprazole	C19 H27 N5	-1.05	9
Cpd 976: Ganoderic acid Mc; C36 H54 O9; 42.647	42.647	630.3769	Ganoderic acid Mc	C36 H54 O9	-0.17	4
Cpd 987: Dofetilide; C19 H27 N3 O5 S2; 42.689	42.689	441.1396	Dofetilide	C19 H27 N3 O5 S2	-0.86	2
Cpd 1075: 5-Hydroxy-7-methoxy-2-tritriacontyl-4H-1-benzopyran-4-one; C43 H74 O4; 46.602	46.602	654.5593	5-Hydroxy-7-methoxy-2-tritriacontyl-4H-1-benzopyran-4-one	C43 H74 O4	-0.9	1
Cpd 1090: Guttiferone A; C38 H50 O6; 48.211	48.211	602.3627	Guttiferone A	C38 H50 O6	-3.24	4
Cpd 1116: His Gln His; C17 H24 N8 O5; 53.345	53.345	420.188	His Gln His	C17 H24 N8 O5	-2.42	4

Figure S7 LC-MS/MS analysis of fraction 3 LC-MS/MS data result of ethyl acetate fraction (fraction 3) presented three highlighted overlapped components; glyceryl 1,3-disterate ($C_{39}H_{76}O_5$), kaempferol 3-O-feruloyl-sophoroside 7-O-glucoside ($C_{43}H_{48}O_{24}$ and hydroxypthioceranic acid ($C_{46}H_{92}O_3$).

Compound Table

Compound Label	RT	Mass	Name	DB Formula	DB Diff (ppm)	Hits (DB)
Cpd 65: Debromoaplysiatoxin; C32 H48 O10; 14.968	14.968	592.3237	Debromoaplysiatoxin	C32 H48 O10	1.84	1
Cpd 75: Neoliquiritin 2"-apioside; C26 H30 O13; 15.207	15.207	550.1685	Neoliquiritin 2"-apioside	C26 H30 O13	0.22	8
Cpd 280: Yuccaol C; C30 H22 O10; 26.553	26.553	542.1223	Yuccaol C	C30 H22 O10	-1.77	3
Cpd 281: Isoneotheaflavin; C29 H24 O12; 26.554	26.554	564.1276	Isoneotheaflavin	C29 H24 O12	-1.46	4
Cpd 382: Gancaonin H; C25 H24 O6; 32.777	32.777	420.1581	Gancaonin H	C25 H24 O6	-1.93	10
Cpd 445: Cassitoroside; C25 H32 O14; 36.074	36.074	556.1789	Cassitoroside	C25 H32 O14	0.5	3
Cpd 485: Hydroxyphthioceranic acid (C46); C46 H92 O3; 36.877	36.877	692.703	Hydroxyphthioceranic acid (C46)	C46 H92 O3	2.4	1
Cpd 489: DG(18:1(11Z)/20:1(11Z)/0:0); C41 H76 O5; 36.976	36.976	648.5705	DG(18:1(11Z)/20:1(11Z)/0:0)	C41 H76 O5	-1.89	10
Cpd 492: Ginsenoside Rh4; C36 H60 O8; 37.072	37.072	620.4292	Ginsenoside Rh4	C36 H60 O8	-0.54	4
Cpd 493: 3-Epipapyriferic acid; C35 H56 O8; 37.074	37.074	604.3999	3-Epipapyriferic acid	C35 H56 O8	-3.97	3
Cpd 515: TG(12:0/13:0/14:1(9Z))[iso6]; C42 H78 O6; 37.320	37.32	678.5808	TG(12:0/13:0/14:1(9Z))[iso6]	C42 H78 O6	-1.44	2
Cpd 527: 1α,25-dihydroxy-26,27-dimethylvitamin D3 / 1α,25-dihydroxy-26,27-dimethylcholecalciferol; C29 H48 O3; 37.437	37.437	444.3603	1α,25-dihydroxy-26,27-dimethylvitamin D3 / 1α,25-dihydroxy-26,27-dimethylcholecalciferol	C29 H48 O3	0.13	10
Cpd 584: Dihydromorelloflavone; C30 H22 O11; 38.475	38.475	558.1174	Dihydromorelloflavone	C30 H22 O11	-2.17	2
Cpd 601: Yuccaol C; C30 H22 O10; 38.708	38.708	542.1208	Yuccaol C	C30 H22 O10	0.85	3
Cpd 606: PA(P-18:0/13:0); C34 H67 O7 P; 38.759	38.759	618.4644	PA(P-18:0/13:0)	C34 H67 O7 P	-3.12	3
Cpd 611: I-Urobilin; C33 H42 N4 O6; 38.872	38.872	590.3107	I-Urobilin	C33 H42 N4 O6	-0.45	2
Cpd 660: Huratoxin; C34 H48 O8; 39.707	39.707	584.3364	Huratoxin	C34 H48 O8	-2.5	3
Cpd 693: Abscisic alcohol 11-glucoside; C21 H32 O8; 40.140	40.14	412.2106	Abscisic alcohol 11-glucoside	C21 H32 O8	-2.09	2
Cpd 730: Cyclohex-1,4-diene-1-carboxyl-CoA; C28 H42 N7 O17 P3 S; 41.267	41.267	873.1584	Cyclohex-1,4-diene-1-carboxyl-CoA	C28 H42 N7 O17 P3 S	-1.48	3
Cpd 761: Kaempferol 3-(2-feruloylsophoroside) 7-glucoside; C43 H48 O24; 42.081	42.081	948.2526	Kaempferol 3-(2-feruloylsophoroside) 7-glucoside	C43 H48 O24	1.04	10
Cpd 772: Pavoninin 1; C37 H59 N O9; 42.389	42.389	661.4194	Pavoninin 1	C37 H59 N O9	-0.61	9
Cpd 782: Ganoderic acid Mc; C36 H54 O9; 42.671	42.671	630.377	Ganoderic acid Mc	C36 H54 O9	-0.33	4
Cpd 786: Dofetilide; C19 H27 N3 O5 S2; 42.711	42.711	441.14	Dofetilide	C19 H27 N3 O5 S2	-1.68	2
Cpd 860: 20:3 Sitosteryl ester; C49 H82 O2; 47.123	47.123	702.6322	20:3 Sitosteryl ester	C49 H82 O2	-1.04	6
Cpd 867: DG(15:0/21:0/0:0)[iso2]; C39 H76 O5; 48.271	48.271	624.5681	DG(15:0/21:0/0:0)[iso2]	C39 H76 O5	1.81	8

Figure S8 LC-MS/MS analysis of fraction 4 LC-MS/MS data result of water fraction (fraction 8) presented three highlighted overlapped components; glyceryl 1,3-disterate ($C_{39}H_{76}O_5$), kaempferol 3-O-feruloyl-sophoroside 7-O-glucoside ($C_{43}H_{48}O_{24}$ and hydroxypthioceranic acid ($C_{46}H_{92}O_3$).

Compound Table

Compound Label	RT	Mass	Name	DB Formula	DB Diff (ppm)	Hits (DB)
Cpd 27: Glucosyl (2E,6E,10x)-10,11-dihydroxy-2,6-farnesadienoate; C21 H36 O9; 12.783	12.783	432.2355	Glucosyl (2E,6E,10x)-10,11-dihydroxy-2,6-farnesadienoate	C21 H36 O9	1.09	1
Cpd 36: 12,14-Nonacosanedione; C29 H56 O2; 13.775	13.775	436.4292	12,14-Nonacosanedione	C29 H56 O2	-2.66	7
Cpd 81: Neoliquiritin 2"-apioside; C26 H30 O13; 15.241	15.241	550.1681	Neoliquiritin 2"-apioside	C26 H30 O13	1.06	8
Cpd 84: 18:2 Sitosteryl ester; C47 H80 O2; 15.374	15.374	676.6135	18:2 Sitosteryl ester	C47 H80 O2	3.46	4
Cpd 388: Yuccaol C; C30 H22 O10; 26.598	26.598	542.1226	Yuccaol C	C30 H22 O10	-2.42	3
Cpd 389: Isoneotheaflavin; C29 H24 O12; 26.606	26.606	564.1281	Isoneotheaflavin	C29 H24 O12	-2.3	4
Cpd 410: Rhazidigenine Nb-oxide; C19 H26 N2 O2; 28.816	28.816	314.1996	Rhazidigenine Nb-oxide	C19 H26 N2 O2	-0.66	1
Cpd 457: 6-Oxocyclohex-1-ene-1-carboxyl-CoA; C28 H42 N7 O18 P3 S; 30.483	30.483	889.152	6-Oxocyclohex-1-ene-1-carboxyl-CoA	C28 H42 N7 O18 P3 S	0.03	1
Cpd 532: Gancaonin H; C25 H24 O6; 32.800	32.8	420.1589	Gancaonin H	C25 H24 O6	-3.75	10
Cpd 607: 1-(8-[5]-ladderane-octanoyl)-2-(8-[3]-ladderane-octanyl)-sn-glycerol; C43 H68 O4; 35.281	35.281	648.5129	1-(8-[5]-ladderane-octanoyl)-2-(8-[3]-ladderane-octanyl)-sn-glycerol	C43 H68 O4	-1.72	1
Cpd 678: Cassitoroside; C25 H32 O14; 36.701	36.701	556.1785	Cassitoroside	C25 H32 O14	1.34	3
Cpd 681: Anastatin B; C21 H14 O7; 36.734	36.734	378.0748	Anastatin B	C21 H14 O7	-2.36	2
Cpd 697: DG(14:1(9Z)/24:1(15Z)/0:0); C41 H76 O5; 36.940	36.94	648.569	DG(14:1(9Z)/24:1(15Z)/0:0)	C41 H76 O5	0.4	10
Cpd 703: 3-Epipapyriferic acid; C35 H56 O8; 37.039	37.039	604.398	3-Epipapyriferic acid	C35 H56 O8	-0.79	4
Cpd 729: TG(12:0/13:0/14:1(9Z))[iso6]; C42 H78 O6; 37.238	37.238	678.5792	TG(12:0/13:0/14:1(9Z))[iso6]	C42 H78 O6	0.97	2
Cpd 832: Dihydromorelloflavone; C30 H22 O11; 38.414	38.414	558.1163	Dihydromorelloflavone	C30 H22 O11	-0.1	2
Cpd 858: PA(P-18:0/13:0); C34 H67 O7 P; 38.703	38.703	618.4632	PA(P-18:0/13:0)	C34 H67 O7 P	-1.26	3
Cpd 926: Huratoxin; C34 H48 O8; 39.655	39.655	584.335	Huratoxin	C34 H48 O8	-0.13	4
Cpd 962: Abscisic alcohol 11-glucoside; C21 H32 O8; 40.084	40.084	412.2098	Abscisic alcohol 11-glucoside	C21 H32 O8	-0.21	2
Cpd 985: Botryococcene; C34 H58; 40.555	40.555	466.4555	Botryococcene	C34 H58	-3.53	2
Cpd 1007: Cyclohex-1,4-diene-1-carboxyl-CoA; C28 H42 N7 O17 P3 S; 41.222	41.222	873.1558	Cyclohex-1,4-diene-1-carboxyl-CoA	C28 H42 N7 O17 P3 S	1.44	3
Cpd 1163: Guttiferone A; C38 H50 O6; 48.199	48.199	602.3624	Guttiferone A	C38 H50 O6	-2.82	4
Cpd 1165: Faradiol laurate; C42 H72 O3; 48.223	48.223	624.5495	Faradiol laurate	C42 H72 O3	-2.16	1
